# Serological, phenotypic and molecular characterization of brucellosis in small ruminants in northern Algeria

**DOI:** 10.3389/fmicb.2024.1505294

**Published:** 2025-01-13

**Authors:** Ibrahim Nabi, Rachid Achek, Abdelkadir Karim, Falk Melzer, Hanka Brangsch, Mandy C. Elschner, Heinrich Neubauer, Hosny El-Adawy

**Affiliations:** ^1^Department of Nature and Life Sciences, Laboratory of Biotechnology and Valorisation of Biological Resources (BVRB), Faculty of Sciences, University Dr. Yahia Farès, Médéa, Algeria; ^2^Department of Biology, Faculty of Nature and Life and Earth Sciences, DjilaliBounaama University, Khemis-Miliana, Algeria; ^3^Laboratory of Food Hygiene and Quality Assurance System (HASAQ), High National Veterinary School, Algiers, Algeria; ^4^Friedrich-Loeffler-Institut, Institute of Bacterial Infections and Zoonoses, Jena, Germany; ^5^Faculty of Veterinary Medicine, Kafrelsheikh University, Kafrelsheikh, Egypt

**Keywords:** brucellosis, *Brucella melitensis*, Algeria, ELISA, whole genome sequencing, small ruminants, SNP typing

## Abstract

Brucellosis is considered a common bacterial zoonotic disease of high prevalence in countries of the Middle East and the Mediterranean region with economic and public health impact. The present study aimed to investigate the current situation of brucellosis in small ruminants reared in Médéa and Sidi Bel-Abbès provinces, north Algeria. To achieve this objective, 96 sera (77 sheep and 19 goat) and 57 milk (42 sheep and 15 goat) samples were collected from suspected infected animals and serologically analyzed by using ELISA. For isolation of *Brucella* spp., four placentas, two fetuses and forty-four milk samples were subjected to microbiological investigation. Whole genome sequencing (WGS) was used for genomic analysis of isolated *Brucella* species. The results of this study showed that anti-*Brucella* antibodies were detected in 46 (83.6%) and 52 (54.2%) milk and serum samples, respectively. However, among 27 cases where blood samples were negative, anti-*Brucella* antibodies were still detected in 19 of the corresponding milk samples, resulting in an overall discordance rate of 36.5%. Ten *Brucella melitensis* were isolated and identified from six sheep and four goats. Of these, eight originated from milk samples. The isolated strains were assigned to sequence type ST-11 using Multilocus sequence typing (MLST). Five isolates revealing high similarity (0–2 nucleotide differences) originated from different farms, indicating a close transmission link. However, two identical caprine isolates and three other isolates showed notable genotypic variation, in comparison. The highest base difference (449–462 nucleotides) was observed for an ovine isolate originating from Sidi Bel-Abbès. The phylogenetic analysis and clustering with the West Mediterranean lineage of *B. melitensis* revealed high genetic similarity of the investigated isolates with *B. melitensis* of human origin from North Africa and travel-associated ‘European’ cases, especially from Morroco, Tunisia, Sweden and Italy. The results of this study highlight brucellosis in small ruminants as a significant public health risk and will help to develop effective control strategies in Algeria. These findings provide specific evidence of this risk, with *Brucella* isolation from milk and by linking theses isolates to human cases in Algeria and abroad. The use of WGS-based analysis has revealed effective in tracing patterns of transmission, and can be recommended for tracking outbreaks at a high resolution.

## 1 Introduction

Brucellosis is a zoonotic bacterial infection caused by members of the genus *Brucella* affecting various animal species. In domestic livestock, the primary species responsible for significant economic losses - due to high rates of abortion and infertility - are *B. abortus* in cattle, *B. melitensis* in sheep and goats, and *B. suis* in pigs (Moreno, 2020). Most human infections are foodborne, i.e., consuming raw milk and unpasteurized dairy products (CDC, 2017). Hence, animal breeders, veterinarians, and dairy or slaughterhouse workers face a high risk of contracting the infection through close contact with infected animals or contaminated materials like placental or foetal tissues. Thus, human brucellosis cases are a clear indicator of the spread of the disease in animals (Moriyón et al., 2023). Despite the successful efforts of eradicating the disease in most European countries, USA, Canada, Australia and others, endemic brucellosis remains a significant health burden in many parts of the world, especially in North and East Africa, the Middle East, Asia and Central and South America (Hull and Schumaker, 2018).

*Brucella melitensis* is the primary aetiologic agent of brucellosis in small ruminants, and it is the most pathogenic brucellae to humans. It causes acute infection of the uterus in sheep and goat ewes resulting in abortion in the mid-third of gestation. For several months, placenta, fetal fluids, and vaginal discharges are the major reservoirs for transmission of the bacterium (Alton et al., 1988). Reinfection often does not cause abortion or severe disease but intermittent/chronic infection of the mammary glands or genital organs (Fensterbank, 1987). Shedding of brucellae with milk and genital secretions by these latent carriers endangers public health (WOAH, 2022).

The prevalence of small ruminant brucellosis in endemic countries may show significant regional differences between administrative districts or geographic regions (Gwida et al., 2010), as the prevalence is depending on the number of livestock as well as eradication programs and strategies implemented by each district and country. The southern part of the Mediterranean basin (Maghreb region) is reported to have the highest incidences in animals and humans (WOAH, 2022; Wareth et al., 2019). As small ruminants constitute the largest livestock group in Algeria (>22 millions of heads), brucellosis in sheep and goats is one of the most important zoonosis. It constitutes an economic burden and a serious hazard for human with a particularly high prevalence compared to neighboring Tunisia and Morocco (Wareth et al., 2019; Kardjadj, 2016).

In Algeria, small ruminant brucellosis was reported for the first time during the colonial period (Sergent, 1908). Imported goats from Malta and Spain were found to be the source of infection. After its independence in 1962, reports on goat and sheep brucellosis cases showed a strong correlation between goat cheese consumption and human brucellosis (Cherif et al., 1986). The first Algerian program to control ruminant brucellosis was launched in 1995 by the Veterinary Services (Inter-ministerial Order of 26 December 1995, official journal N°65. 30-10-1996) and made use of serological screening and animal slaughter. However, this policy has failed to control the disease because of neglecting essential factors. First, pastoralist breeding involves free movement of flocks across vast areas (for grazing) during hivernal and estival seasons, even across province borders. This practice obscures the exact localization of outbreaks, increases transmission risk and impairs traceability. In addition, the absence of animal or herd identification system makes it difficult to differentiate previously aborted animals from healthy ones. Finally, slaughter penalties combined with a weak insurance system discourage the breeders from reporting suspected cases and coopering with officials (Tazerart et al., 2022). In 2002, the National Veterinary Medicine Institute (INMV) estimated herd prevalence at 5.68 to 10% in the steppe region [reviewed by (Kardjadj, 2016)]. In 2004 to 2006, the individual prevalence in goats was found to be 13.41% in ten provinces (Lounes and Bouyoucef, 2008). Facing this situation, the Algerian authorities introduced a large-scale *B. melitensis* Rev-1 vaccination campaign as a new prophylactic approach in 2006 starting with the steppe region. Subsequently, the official herd prevalence of small ruminant brucellosis had decreased to 3.3% in 2014, (Kardjadj, 2016). However, in 2016, the vaccination campaign for small ruminants was terminated.

The epidemiological link between *Brucella* strains currently circulating in Algeria remains unclear. The majority of recently published findings on sheep and goat brucellosis has been obtained from routine seroprevalence surveys using classical serological tests (Haif et al., 2021). Varying prevalences at flock level were found in different studies and regions: 7.7–17.5% in the West (Rechidi-Sidhoum et al., 2018); 39.1% in arid zones (Kouri et al., 2018) and 27.9% in the Southeast (Ramdani et al., 2022). Several case reports have documented that Algerian emigrants have contracted brucellosis after consuming unpasteurized goat or sheep milk products during their stay in their country of birth (Benammar et al., 2022; Blanc-Gruyelle et al., 2017; Bréhin et al., 2016; de Nettancourt et al., 2022; Kitt et al., 2017).

Previous studies on small ruminant brucellosis in Algeria were limited in investigating brucellosis comprehensively in herds with a history of abortions during the lambing period or in relation to the reproductive status of ewes. Notably, only one study reported 26 *B. melitensis* strains isolated from 38 samples gained from goat abortion, proving the involvement of *Brucella* in small ruminant miscarriages (Gabli et al., 2015). Although milk consumption is strongly connected to the epidemiology of human brucellosis (Benammar et al., 2022; Blanc-Gruyelle et al., 2017; Bréhin et al., 2016; de Nettancourt et al., 2022; Kitt et al., 2017), there has been a lack of investigations studying *Brucella* prevalence in milk using ELISA, molecular technologies or bacterial isolation.

This study aimed to investigate potential *Brucella* infection in small ruminants (sheep and goats) during the reproductive period after abortions or postpartum complications using serological, microbiological and molecular biological tools.

## 2 Materials and methods

### 2.1 Study description and sample collection

**Figure 1:**
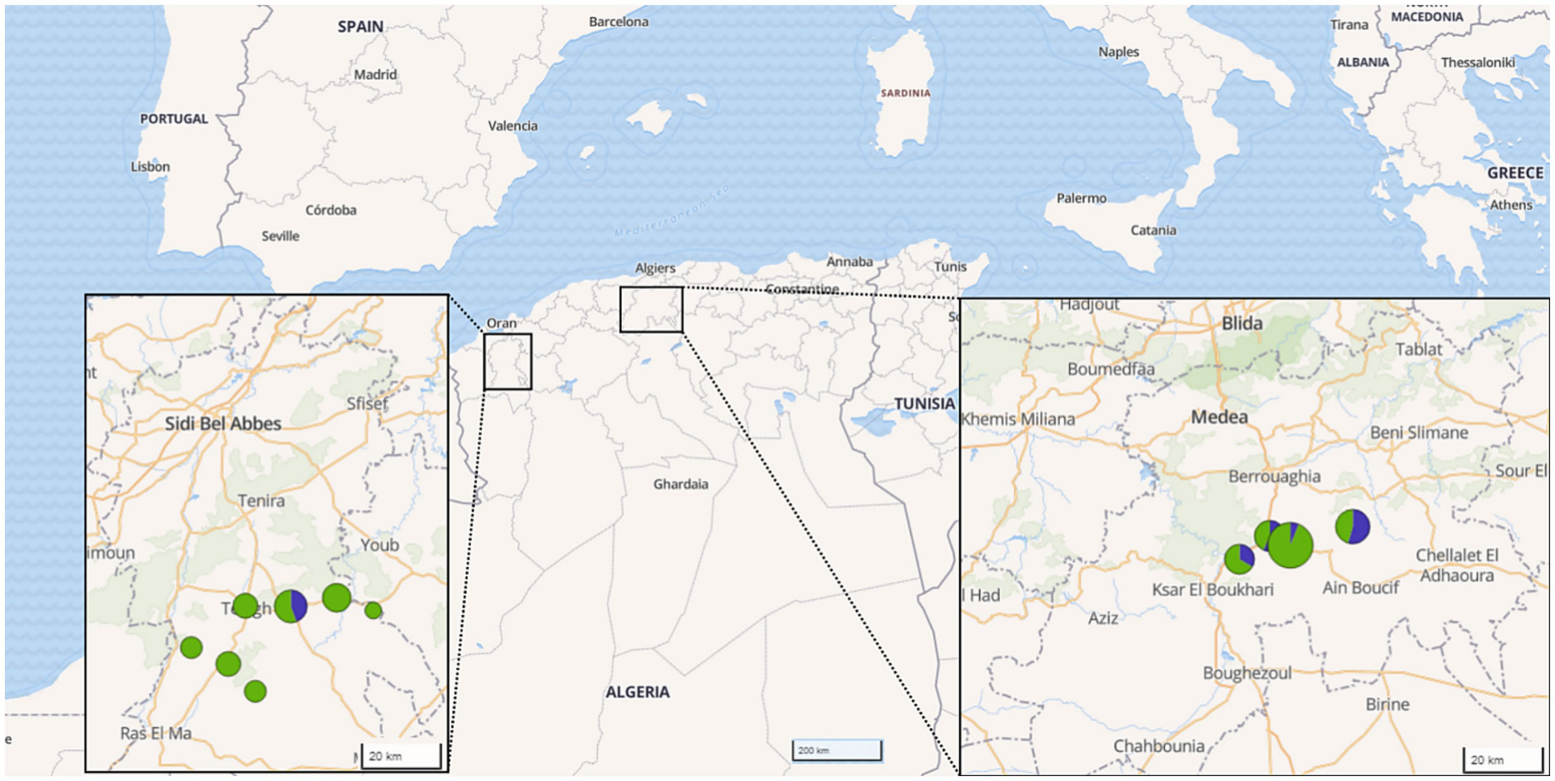
Origin of samples investigated by provinces in Algeria. The color and size of the circles indicate the number of samples per species and region (green: sheep, blue: goat).

From March 2020 to January 2022, a total of 25 farms across 11 different regions in the provinces of Médéa and Sidi Bel-Abbès, north Algeria, were sampled. The chosen regions were known for small ruminant breeding, with an estimated capacity of 875,000 ovine heads (MADR, 2021), and both are part of the agropastoral southern band of the Atlas mountains. The selection of these regions was based on prior reports on high incidence of brucellosis-associated abortion and human cases. The selected farms represented a cross-section of the affected areas. They were identified in collaboration with local veterinarians and farmers who had observed clinical reproductive disorders - mainly abortions - potentially related to brucellosis. Thus, all 25 farms were suspected of brucellosis. The studied farms had, on average, 89 heads of local breed, reared under a traditional semi-intensive agropastoral breeding system. The sheep and goats within each herd were co-mingled and grazed on free fallow pasture, often interacting with other pastoralist herds coming from southern steppe provinces, during the transhumance period. All selected herds were suffering from signs of brucellosis, e.g., abortion, premature birth, placental retention, mastitis and morbid stillbirth. The sampling priority was given to animals for which reproductive problems have been reported recently. In total, 80 sheep and 19 goats (Table 1 and Figure 1), with an average age of 3.6 years and no history of previous vaccination against *Brucella*, were sampled during the lambing season in September to December of each year.

**Table 1:** Numbers and geographical origin of sera and milk samples from sheep and goats.

Provinces	Regions	Farms ID	cases	No. of serum		No. of milk	
				Sheep	Goat	Sheep	Goat
Sidi-Bel-Abbès	Ain-Tindamin	22.F03	2	2	0	2	0
	Oualla	22.F05	1	1	0	1	0
	Dhaya	22.F02	3	0*	0	3	0
	Merin	22.F01	2	1	1	1	1
		22.F06	1	1	0	1	0
		22.F08	3	3	0	-	-
		22.F10	1	0	1	0	1
		22.F11	2	0	2	0	2
	Sidi-Chaib	22.F07	2	2	0	2	0
	Tawrira	22.F04	5	5	0	5	0
	Tlagh	22.F09	3	3	0	3§	0
	**Total**	**11 farms**	**25**	**18**	**4**	**18**	**4**
Médéa	Moudjebour	26.F08	4	3	1	0	1§
		26.F09	1	1	0	-	-
		26.F14	1	0	1	0	1
	Rebaia	26.F01	6	0	6	0	3
		26.F07	1	1	0	-	-
		26.F12	4	4	0	4	0
	Seghouane	26.F13	7	3	4	3	4
	Tlatet Eddouair	26.F02	19	16	3	9	2
		26.F03	20	20	0	5	0
		26.F04	1	1	0	-	-
		26.F05	1	1	0	-	-
		26.F06	1	1	0	-	-
		26.F10	4	4	0	-	-
		26.F11	4	4	-	3	-
	**Total**	**14 farms**	**74**	**59**	**15**	**24**	**11**

^*^Serum samples were lost; ^§^Milk clumps, cannot be pipetted / used for ELISA.

For each animal, blood was collected in plain Vacutainer® tubes, labeled and transported on ice to the laboratory. The sera were then separated from the clot by centrifugation and stored at −20°C until analysis. If milk was present in the udder, a sample was taken in an aseptic container and frozen at −20°C. In cases where an on-site abortion was observed, samples of the aborted tissues were collected aseptically. Along with the collection, each animal was assigned an identification number (ID No), and several risk factors were recorded, including age, parity, lactation status, history of abortions, history of placental retention, presence of mastitis, and timing of sampling after parturition or abortion. In total, 96 sera, 57 milk, 4 placental tissues and 2 foetal tissues were collected and shipped to the Friedrich-Loeffler-Institut for serological, microbiological and genotyping analysis.

### 2.2 Detection of anti-*Brucella* antibodies in serum and milk

Serum and milk samples were screened by ID Screen® Brucellosis Serum Indirect Multi-species (IDVet®, Grabels, France) and ID Screen® Brucellosis Milk Indirect (IDVet®, Grabels, France), an indirect-ELISAs for detection of anti-smooth-lipopolysaccharide (LPS) (*B. abortus*, *B. melitensis* and *B. suis*) according to the manufacturer’s recommendations. The diagnostic sensitivity of iEISA were shown to be comparable to other certified commercial ELISA 0.942–1.0 and specificity 0.906–1.0 (García-Bocanegra et al., 2014).

### 2.3 Bacterial isolation

Placenta (*n* = 2), mastitis milk (*n* = 4), foetal tissue (*n* = 2) and milk (*n* = 44) samples of seropositive animals were cultured using classical method. Briefly, tissue samples were manually homogenized by mortar and pestle grinding under sterile conditions. Approximately 10 μL of each homogenate was used for inoculation, while milk samples were streaked directly. Inoculates were plated first on blood agar (7.5% calf blood) and *Brucella* agar (Oxoid Deutschland GmbH, Wesel, Germany) for isolation. Single colonies with characteristic growth were sub-cultered again on *Brucella* selective agar to obtain uncontaminated isolates. All plates were incubated with 5 to 10% CO_2_ at 37°C. Plates were observed for bacterial growth on the 3rd, 5th and 7th day. Suspected colonies were subjected to Gram staining, oxidase and catalase tests, as well as mobility testing (Alton et al., 1988).

### 2.4 Species identification by Bruce-ladder multiplex PCR

Genomic DNA was extracted by using HighPure PCR Template Preparation kit (Roche® Germany) according to the manufacturer’s instructions. Species identity was confirmed by Bruce-ladder multiplex PCR as described previously (López-Goñi et al., 2008; García-Yoldi et al., 2006).

The major advantage of this assay over previously described PCRs is that it can identify and differentiate in a single step most *Brucella* species as well as the vaccine strains *B. abortus* strain 19 (S19), *B. abortus* RB51 and *B. melitensis* Rev.1. In contrast to other PCRs, Bruce-ladder is also able to detect DNA from *B. neotomae*, *B. pinnipedialis* and *B. ceti*. An updated multiplex PCR assay (Suis-ladder), has been developed for fast and accurate identification of *B. suis* strains at the biovar level (WOAH, 2022; López-Goñi et al., 2011).

### 2.5 Whole genome sequencing and bioinformatic analysis

Sequencing library preparation was done with the Nextera XT library prep kit (Illumina Inc., San Diego, CA, United States) and the libraries were sequenced on a MiSeq (Illumina Inc., San Diego, CA, USA) sequencer in paired-end mode. Following quality control of the raw reads using FastQC v0.12.1 (Andrews, 2010), genomes were assembled by Shovill v1.0.4 (https://github.com/tseemann/shovill) and checked by QUAST v5.2.0 (Gurevich et al., 2013). The assemblies were subjected to *in silico* 9-loci multilocus sequence typing (MLST-9) using the tool mlst v2.23.0 (https://github.com/tseemann/mlst) with the scheme available on PubMLST (Jolley et al., 2018). Further, single nucleotide polymorphism (SNP) typing was done using Snippy v4.6.0 (https://github.com/tseemann/snippy) with *B. melitensis* 16 M (GCF_000007125.1) as reference genome for determining the *B. melitensis* lineage that the isolates belong to and *B. melitensis* Ether (GCF_000740355.1) as reference for detailed genotyping. SNP differences were counted by the script snp-dists v0.8.2 (https://github.com/tseemann/snp-dists). Foreign Illumina read data from *B. melitensis* strains from different lineages, the Mediterranean Basin and suspected human cases from Europe were downloaded from NCBI’s Short Read Archive (accession: 13.05.2024; Supplementary Table S1) and included in the SNP analysis. The SNP alignment was analyzed by Maximum Likelihood analysis using RAxML v8.2.12 (Stamatakis, 2014) and the tree visualized by Microreact (Argimón et al., 2016), which was also used for creating a map. The sequencing raw data was uploaded to the European Nucleotide Archive (ENA) and is publicly available under the BioProject number PRJEB76942.

### 2.6 Statistical analysis

Descriptive analysis and statistical tests were done using IBM SPSS Statistics v25 2015 (IBM Corp., Armonk, NY, United States). Correlation of potential risk factors (geographical location, specie, sex, age group, parity, herd size, gestation status, history of sampling and previous abortion) with serology results of 96 serum samples and 55 milk samples was analyzed using Pearson’s Chi-squared test (X^2^), Fisher’s exact test (when expected values are <5) and odds ratio (OR).

## 3 Results

### 3.1 Prevalence of anti-*Brucella* antibodies in sera and milk samples

From a total 96 samples, 52 (54.2%) sero-positive samples harbored anti-*Brucella* spp. antibodies, that were detected using the i-ELISA technique (Table 2). The seropositivity of goats was higher (63.2%) than that detected in sheep (51.9%). Out of 93 investigated ewes, 50 (53.8%) were tested positive. Two out of three rams suspected of brucellae transmission were tested positive. This study analyzed 55 milk samples from lactating females. Forty-six (83.6%) were found positive. Sheep and goat milk samples displayed similar prevalence (82.9 and 85.7%, respectively).

**Table 2:** Detection of anti-*Brucella* antibodies in serum and milk samples using ELISA.

**Variable**	**Level**	**Blood**				**Milk**			
		**Total**	**positive n (%)**	***X***^**2**^ **Fisher’s**^**a**^	***p*-value**	**Total**	**Positive *n* (%)**	***X***^**2**^ **Fisher’s**^**a**^	***P*-value**
Province	Médéa	74	46 (62.2)	8.31	0.004	34	32 (94.1)	7.148	0.008
	Sidi Bel-Abbès	22	6 (27.3)			21	14 (66.7)		
Region	Moudjebour	5	0 (0)	29.7^a^	0.0001	1	1 (100)	12.94^a^	0.069
	Rebaia	11	3 (27.3)			7	7 (100)		
	Seghouane	7	7 (100)			7	7 (100)		
	Tlatet Eddouair	51	36 (70.6)			19	17 (89.4)		
	Ain-Tindamin	2	1 (50)			2	1 (50)		
	Oualla	1	0 (0)			1	0 (0)		
	Dhaya	-	-			3	2 (66.7)		
	Merin	9	4 (44.4)			6	4 (66.7)		
	Sidi Chaib	2	0 (0)			2	2 (100)		
	Tawrira	5	1 (20)			5	3 (60)		
	Telagh	3	0 (0)			2	2 (100)		
Specie	Sheep	77	40 (51.9)	0.771	0.38	41	34 (82.9)	0.059	0.8
	Goat	19	12 (63.2)			14	12 (85.7)		
Age group	< 2 Years	22	13 (59.1)	13.22^a^	0.003	7	4 (57.1)	11.85^a^	0.003
	2–4 Years	46	19 (41.3)			20	14 (70)		
	4–6 Years	23	19 (82.6)			24	24 (100)		
	> 6 Years	5	1 (20)			4	4 (100)		
Herd size	< 50 heads	28	16 (57.1)	7.341^a^	0.054	19	18 (94.7)	3.25^a^	0.338
	50–100 heads	26	19 (73.1)			10	8 (80)		
	100–150 heads	38	16 (42.1)			22	17 (77.3)		
	> 150 heads	4	1 (25)			4	3 (75)		
Previous abortion	0	36	16 (44.4)	5.989^a^	0.083	27	20 (74.1)	2.068^a^	0.389
	1	38	17 (44.7)			20	18 (90)		
	2	10	8 (80)			3	3 (100)		
	3	2	2 (100)			-	-		

^*^The time point of sample collection relative to parturition or abortion.

### 3.2 Associated risk factor analysis

The analysis of various risk factors (Table 2) revealed an association between seropositivity and animal’s age. Specifically, animals in the 2–4 and 4-6-years age groups had the highest prevalence (38/96, 39.5%, altogether) (*p* = 0.003). 13/20 (65%) of females with natural parturition and 31/64 (48.4%) of females with abortion were tested positive. Hence, a clear trend was observed toward an abortion history (*p* = 0.083); females with two or three previous abortions had sero-prevalences of 80 and 100%, respectively. Interestingly, the region factor was found to be significantly associated with *Brucella* seropositivity (*p* = 0.004). The odds ratio (OR) of 0.228 indicated that animals of herds with abortion history in the Médéa province were significantly more likely to be seropositive (62.2%), compared to similarly affected herds in the Sidi Bel-Abbès province (27.3%).

Consistent with findings in blood serum analysis, milk samples from females aged 2–4 and 4-6-years exhibited significantly (*p* = 0.003) higher prevalence (70 and 100%, respectively). The regional factor emerged to have significant association with antibody detection in milk, (*p* = 0.008). Milk samples from the Médéa region showed a near eightfold increase in risk (odds ratio = 0.125) and exhibited higher positive case proportion (94.1%) compared to those sampled from Sidi Bel-Abbès (66.7%).

### 3.3 Cross-outcomes with serum and milk serology

Out of 52 cases, 25 (48.1%) blood serum ELISA positive samples were found also positive by milk ELISA. Over a third (19 of 52, 36.5%) of females tested serum-negative, were found positive in milk ELISA, demonstrating discordant results, and nearly half of these “milk-positive, serum-negative” females had experienced a previous abortion.

Furthermore, the double-negative ewes (negative in both serum and milk) were markedly younger (mainly from age group 2–4 years) compared to the majority of positive cases. A previous abortion history was documented for nearly half of the positive cases (both double positive and milk-positive only) (Table 3).

**Table 3:** Cross outcomes of anti-*Brucella* antibodies in blood and milk samples and correlation to abortion history.

ID-Vet® Serology			Previous abortions			
Serum	Milk	In total	0	1	2	ND
Negative n (%)	Negative	8 (15.4)	6 (24)	2 (10.5)	0 (0)	–
	Positive	19 (36.5)	9 (36)	9 (47.3)	1 (33.3)	–
**Sub total**		27 (51.9)	15 (60)	11	1	-
Positive *n* (%)	Negative	–	–	–	–	–
	Positive	25 (48.1)	10 (40)	8 (42.1)	2 (66.6)	5 (100)
**Total**		52	25	19	3	5

### 3.4 Phenotypic characterization of cultured samples

Ten isolates were obtained (Table 4) that displayed characteristic features of *Brucella.* The Bruce-ladder PCR revealed that all ten isolates were *Brucella melitensis*. Nine of the ten isolates originated from Médéa province, whereas isolate 23RB26678 was from Sidi Bel-Abbès province in northwestern Algeria.

**Table 4:** Metadata of *Brucella* isolates isolated from different matrices and their SNP type (i.e., strains with SNP distances less than two SNPs).

Strain	Host	Date	Region	Herd ID	Source	Identification	SNP type
23RB25956	Sheep	11.2021	Tlatet Eddouair	26.F05	Placenta	*Brucella melitensis*	1
23RB25957	Sheep	12.2021	Tlatet Eddouair	26.F02	Mastitis	*Brucella melitensis*	1
23RB25958	Sheep	12.2021	Tlatet Eddouair	26.F02	Foetus	*Brucella melitensis*	1
23RB26011	Sheep	11.2021	Tlatet Eddouair	26.F02	Milk	*Brucella melitensis*	1
23RB26012	Goat	03.2021	Rebaia	26.F01	Milk	*Brucella melitensis*	–
23RB26013	Goat	12.2021	Moudjbeur	26.F14	Milk	*Brucella melitensis*	–
23RB26256	Goat	01.2022	Seghouane	26.F13	Milk	*Brucella melitensis*	2
23RB26257	Goat	01.2022	Seghouane	26.F13	Milk	*Brucella melitensis*	2
23RB26679	Sheep	01.2022	Seghouane	26.F13	Milk	*Brucella melitensis*	1
23RB26678	Sheep	03.2020	Dhaya	22.F02	Milk	*Brucella melitensis*	–

### 3.5 Genotyping of isolated *Brucella*

**Figure 2:**
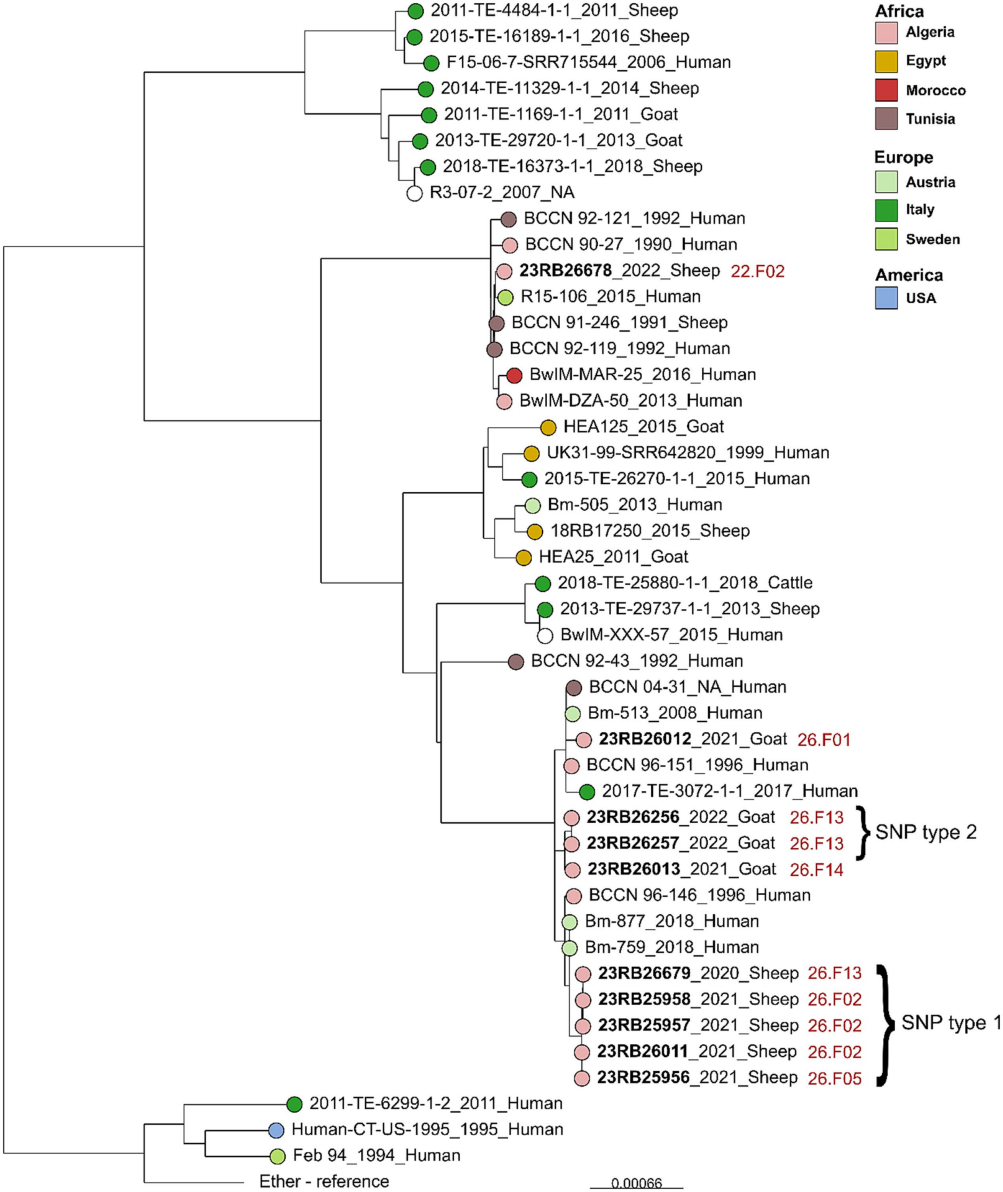
Maximum likelihood tree based on core genome SNP alignment of *Brucella melitensis* strains of the West Mediterranean lineage. Printed in bold are the isolates from the current study with farm IDs indicated in red with name, date and source of isolation. The bar indicates nucleotide changes per site of the alignment.

Genome sequencing and assembly yielded genomes of the expected size (mean: 3.3 Mb) and GC content (57.2%) for *B. melitensis* (Supplementary Table S2). The *in silico* MLST analysis showed that all isolates belonged to sequence type (ST) 11 and SNP typing assigned the isolates to the West Mediterranean lineage of *B. melitensis*. To achieve a higher level of discrimination between the sequenced isolates for outbreak tracing and to explore similarities with foreign strains, the cgSNP typing approach was employed. The difference between five ovine isolates (23RB25956, 23RB25957, 23RB25958, 23RB26011, 23RB26679) was found to be 0–2 SNPs, wherefore they were assigned to the same SNP type (type 1, Table 4). These isolates originated from three different farms in two regions of Médéa. Likewise, two caprine isolates (23RB26256, 23RB26257) from the same farm were identical in SNP typing and were designated SNP type 2. Interestingly, this farm was also the origin of isolate 23RB26679 that belonged to SNP type 1, showing that two different *B. melitensis* SNP types were found on the same farm. These two SNP types were quite distinct from each other displaying 53–55 base substitutions. The isolate 23RB26678 from a sheep in Dhaya, a commune of Sidi Bel-Abbès province, showed the most genotypic differences to the other nine sequenced isolates (449–462 SNPs difference). When compared to other *B. melitensis* strains of the West Mediterranean lineage from North Africa and travel-associated ‘European’ strains, it was found that the isolate from Sidi Bel-Abbès was highly similar to human strains from Sweden, Algeria and Morocco (16–28 SNPs) isolated between 1990 and 2016 (Figure 2). The other Algerian isolates also clustered with isolates from human brucellosis cases found in Austria showing 11–12 and 28–29 SNP differences compared to SNP types 1 and 2, respectively. The analyzed isolates further clustered with strains from Italy and Tunisia. The two SNP types formed two separate clusters on that branch.

## 4 Discussion

In Algeria, small ruminant brucellosis remains a persistent enzootic threat despite extensive *Brucella* control efforts including a large vaccination campaign from 2006 to 2016 (Kardjadj, 2016; Lounes et al., 2014). Previous serological studies have shed light on *Brucella* prevalence revealing regional variations (from 5 to 16%) (Kardjadj, 2016; Rechidi-Sidhoum et al., 2018; Gabli et al., 2015). Currently, private veterinarians and breeders realize an increase of the number of abortions in sheep and goats. Human brucellosis cases due to close contact with animals or the consumption of unpasteurized milk underline the urgency of the need for action (de Nettancourt et al., 2022; Kitt et al., 2017). Surprisingly, there are only few studies on brucellosis in flocks being suspected of brucellosis or in the postpartum period in general. Thus, there is a critical gap in knowledge for data concerning the actual bacterial prevalence and consequently on the molecular identity of circulating *Brucella* isolates.

Thus, our study included serological investigations on ewes and female goats with abortion or suspected brucellosis by using ELISA, as well as whole genome sequencing with subsequent core genome SNP typing of *Brucella* strains. We investigated brucellosis in suspected flocks in two regions, the province of Médéa in the North Centre and the province of Sidi Bel-Abbès, in the North West of Algeria. These two regions are known as transitional zones between Tell Atlas and the pastoral steppe areas. Médéa and Sidi Bel-Abbès were chosen as study regions based on several records of abortions or suspected brucellosis cases. The design of our preliminary study was limited by the restricted study area and low sample size, and does not allow generalized conclusions about the prevalence of brucellosis in all small ruminant herds. However, data from Médéa region confirmed a steadily high level of infection, as previous surveys have found a high seroprevalence of brucellosis in small ruminants in the north Centre, i.e., Médéa and neighboring regions (31% in goat herds) (Lounes and Bouyoucef, 2008). Regardless, only one previous study on *Brucella* isolation and characterization in the same region was addressed in cattle slaughter houses of Médéa, covering only two regions (Khames et al., 2017). Although there were no published records on animal brucellosis in Sidi Bel-Abbès, we expected the occurrence of brucellosis for this region as seroprevalences of 7.7 and 17.5% were reported in sheep and goat herds, respectively, for the neighboring region of Mostaghanem (127 km away) (Rechidi-Sidhoum et al., 2018). However, the unexpectedly high prevalence indeed requires immediate investigations to tailor adequate countermeasures to reduce the risk for human infection and losses to the owners. The limited sample size and the regional focus limit the ability to capture the full genetic diversity of *Brucella* strains across Algeria and may not fully represent transmission dynamics in other parts of the country, especially in areas with different animal movement patterns and epidemiological risks.

Previous studies reported that multiparous goats were more likely to be seropositive than primiparous (Gabli et al., 2015). This finding is not surprising because older animals have a higher risk of exposure in an endemic area. The present study confirmed this finding again, as older females tested more often positive in both serum and milk samples. It was also speculated that in younger small ruminants *Brucella* infections are less common due to the lack of sex steroid hormones and erythritol in the uterus, which stimulate the growth and multiplication of *Brucella* organisms (Radostits et al., 2007).

The present findings showed a strong correlation between history of abortion and positive samples. Furthermore, all *Brucella* isolates belonged to ewes with abortion history that were double positive in serum and milk. These findings were in concordance with a previous study conducted on nomadic goat herds of the east Algerian steppe showing a high relation between abortion cases and isolation of *B. melitensis* bv3 (68%) (Gabli et al., 2015). Recently, in southeast of Algeria, another study showed the same trend (Ramdani et al., 2022).

It is known that *Brucella*-related abortion in ewes during the mid-third of gestation in primo-infection coincides with massive invasion of the bacterium and fever (CDC, 2017).

To the knowledge of the authors, this is the first Algerian study using milk serology on samples from brucellosis suspected small ruminants. Various studies confirmed a high risk for humans in Algeria to get infected when consuming milk products especially those made from goat milk (Benammar et al., 2022; Blanc-Gruyelle et al., 2017; Bréhin et al., 2016; de Nettancourt et al., 2022; Kitt et al., 2017). Thus, it is not surprising to find a high rate (84.6%) of ELISA positive milk samples in this preliminary study concentrating on herds with clinical symptoms suspected for brucellosis. This working hypothesis is also the obvious reason for the perfect concordance between positive serum samples, positive milk samples and positive culture. In Yemen, the seroprevalence in milk from small ruminant with abortions was investigated and showed 49 and 41.2% of milk samples from sheep and goats were positive, respectively (Al-Afifi et al., 2022). Interestingly, we found that 36% of blood serum negative ewes were milk positive. This discordance appeared in older ewes regardless of their specific reproduction status. In agreement with a former study in India, out of 250 milk and 250 blood samples of diseased goats, 34.8% of milk samples but only 16% serum samples were tested positive for brucellosis (Lonkar et al., 2023).

The milk i-ELISA test could be used as a rapid screening test for brucellosis in unvaccinated dairy cows (Wang et al., 2020). The discordances between blood and milk serum results could be explained, on one hand by the fact that although i-ELISA has a high specificity and sensitivity, seropositive outcomes could be associated with a cross-reaction caused by other bacterial LPS, including *Salmonella* sp.*, Escherichia coli, Escherichia hermannii, Yersinia enterocolitica,* and *Vibrio cholerae* (Lord et al., 1998). On the other hand, animals in endemic areas which are in close contact with brucellae could develop a long lasting memory response against this antigen through only local immunological response of the mama (Herrera-López et al., 2010).

*Brucella* isolation by culturing remains the gold standard for brucellosis diagnostics. In the present study, bacteriological investigations of samples from highly suspected cases have led to the isolation of 10 *Brucella melitensis* strains. It is known that *B. melitensis* is the main causative agent of brucellosis in small ruminants (Rossetti et al., 2022), and that *B. melitensis* bv 3 is the predominant biovar isolated independent of the host species in Egypt (Wareth et al., 2014). In a previous study in Algeria, 26 *B. melitensis* were isolated from vaginal swabs of 38 goat abortions (Gabli et al., 2015). These findings show that the time of sampling (abortion, lambing) is crucial for a positive culture and that a high risk of brucellosis infection is encountered from milk consumption. Milk is a very good matrix for brucellae survival, as the bacteria may survive in milk for 130 days (Falenski et al., 2011; Kaden et al., 2018).

Whole genome sequencing-based typing methods offer superior discriminatory power for differentiating closely related strains and allow for detailed core genome SNP-based comparative analysis which is an accurate approach for epidemiological investigations (Whatmore and Foster, 2021). This study is the first in Algeria applying WGS technology for providing insights into the genomic diversity of brucellae that caused infections in small ruminant. *In silico* MLST analysis and SNP typing revealed that all isolates belonged to ST 11 within the West Mediterranean lineage of *B. melitensis*. Previous findings on molecular typing using MLVA indicated that most of Algerian brucellae from humans and cattle also belonged to this group with similarity to European and Maghreb strains (Lounes et al., 2014; Khames et al., 2017).

In Médéa province, all sheep isolates from three different farms belonged to a single cluster with low SNPs difference suggesting a common source of infection and a close connection in the transmission of brucellosis. Identical SNP profiles among certain isolate pairs indicate the spread of the same strains, probably through shared animal movements or use of the same breeding rams. These farms are geographically close, share a common grazing area and engage in frequent bilateral trades.

In contrast, *B. melitensis* strains isolated from goats were found distinct (53–58 SNPs) from those isolated from sheep. This was observed on farm 26.F13 where caprine strains were SNP type 2 and ovine strain was SNP type 1, which indicates potential repeated introductions from diverse sources. Remarkably, when comparing SNP differences among caprine strains, the minimal SNPs distance (18 SNPs) was observed between strains from farm 26.F13 and neighboring farm (26.F14) in Moudjbeur region. Moudjbeur, known for goat breeding, has recorded several human brucellosis cases primarily linked to goat milk consumption (unpublished records), suggesting an epidemiological link related to these two farms. The agro-pastoral breeding system in the studied and neighboring regions facilitates unrestricted herd movements of local or transhumant farmers, bilateral trade, and therefore potential disease transmission. Local livestock markets could be focal points of spread where close contact can facilitate potential disease transmission. Further studies are needed on molecular level to understand disease patterns and transmission pathways.

SNP typing is a high-resolution tool for differentiating *Brucella* strains and allows the detection of small genetic variations, thereby enabling tracing the sources of infections. This powerful tool helps researchers to understand transmission pathways between different hosts and geographic regions and is especially useful for pinpointing cross-species transmission events.

The core genome SNP comparison to other *B. melitensis* strains of the West Mediterranean lineage revealed that most of studied isolates (from Médéa province) closely clustered with isolates from human brucellosis cases in Tunisia, Italy and Austria. Based on publication data, the strains from Austria originated from two veterinarians (Bm-877 and Bm-759) and another patient (Bm-513) (Schaeffer et al., 2021) representing two outbreak events in 2018 and 2008, respectively. The high similarity between the here investigated isolates and those Austrian strains (11–12 SNPs and 22–31 SNPs difference, respectively) suggests an epidemiological link. These observations rise important concerns on the transmission paths along Algeria-Tunisia-Italy up to central Europe. The incrimination of brucellosis transmission via importation of infected goats from Italy was historically reported (Wareth et al., 2019). Previous studies showed increased prevalence of brucellosis, that could also be proven by *Brucella* isolation, among humans and clinical abortions in ewes from municipalities near to the Tunisian border (Ramdani et al., 2022; Benammar et al., 2022; Gabli et al., 2015). This potential cross-border transmission could result from limited control efforts, uncontrolled movement of animals across borders, traditional milk consumption and the nomadic farming system.

The only isolate from Sidi Bel-Abbès (west Algeria) (23RB6678) clustered with strains from Morocco (BwIM-MAR25) (Georgi et al., 2017), Tunisia and Sweden (R15-106). The latter was from an Eritrean immigrant to Sweden and thought to be of Moroccan origin based on WGS analysis (Sacchini et al., 2019). In the SNP analysis 23RB6678 markedly differed from studied stains isolated in central Algeria. This observation could hint at possible transmission routes across the western border of neighboring countries. Connections between brucellosis outbreaks in Algeria and Morocco have previously been reported (Sergent, 1908). Bilateral trading and illegal uncontrolled movement of small ruminants could constitute a potential transmission pathway.

Altogether, the present study highlighted clustering patterns of strains from similar geographical origin, that prompts us to assume a geographical and socioeconomic link of brucellosis transmission between countries of both sides of the Mediterranean Sea. Risk factors such as transhumance, importation of infected animals, uncontrolled movement across borders and consumption of contaminated milk products by individuals visiting endemic regions are well known (Tazerart et al., 2022; de Nettancourt et al., 2022; Gabli et al., 2015) and have led to international spread of *Brucella* lineages in the past. The current findings highlight also the deficiencies in the existing surveillance program and the limited effectiveness of current sensitization policies for controlling brucellosis. Effective and updated recommendations based on advanced diagnostic and surveillance technologies should be urgently communicated to veterinary authorities to strengthen preventive measures. However, further studies that include large number of cases, complete epidemiological data and environmental as well as animal samples are needed to draw meaningful conclusions.

## 5 Conclusion

The results of this study highlight brucellosis in small ruminants exhibiting reproductive disorders as a significant public health risk. To the best of our knowledge, the present study provides the first in-depth serological, microbiological and molecular investigation of small ruminant brucellosis in Algeria. The full genome sequencing of isolated *Brucella* in this study revealed that all isolates belonged to ST-11 which is a dominant lineage in North Africa. The core genome SNP analysis brings valuable insights into spatial distribution of regional outbreaks and reveals close clustering with human strains from Maghreb and European countries. The geographical transmission patterns across western and eastern borders, uncontrolled movement of animals and immigration along with consumption of local contaminated foods or illegal import of dairy products are the major risk factors for brucellosis dissemination both domestically and internationally. Evidence of *Brucella* shedding in the milk of sheep and goats demonstrated the high risk for spread of the disease and a serious public health hazard within Algeria and abroad.

The present findings highlight the importance of implementing validated milk serology as part of routine screening of small ruminants. Strengthening biosecurity protocols, especially at national borders, through inspection and health certification based on reliable serology for transported livestock is recommended and can mitigate the spread of *Brucella* across regions. The development of public awareness campaigns to educate rural communities on the risks associated with brucellosis and the dangers of un-pasteurized dairy products consumption must be given great attention. The present findings can be considered a base-line to conduct larger studies that provide a clear understanding of transmission dynamics of brucellosis infections in Algeria. The use of WGS-based analysis has revealed effective in tracing patterns of transmission, and can be recommended for tracking outbreaks at a high resolution. The integration of SNP analysis with coordinated epidemiological data sharing between neighboring countries, e.g., information on livestock grazing and trade routes or human travel, enables the identification of transmission routes and contributes to designing control measures.

Based on the outcomes of the present study, the finding of this study will help to develop effective control strategies in Algeria especially in endemic regions.

## Data Availability

The datasets presented in this study can be found in online repositories. The names of the repository/repositories and accession number(s) can be found in the article/Supplementary material.
